# A Metamaterial Inspired AMC Backed Dual Band Antenna for ISM and RFID Applications

**DOI:** 10.3390/s22208065

**Published:** 2022-10-21

**Authors:** Md. Najumunnisa, Ambadapudi Srinivasa Chandrasekhara Sastry, Boddapati Taraka Phani Madhav, Sudipta Das, Niamat Hussain, Syed Samser Ali, Muhammad Aslam

**Affiliations:** 1 Department of Electronics and Communication Engineering, Koneru Lakshmaiah Education Foundation, Vaddeswaram 522302, India; 2 Department of Electronics and Communication Engineering, IMPS College of Engineering and Technology, Malda 732103, India; 3 Department of Smart Device Engineering, Sejong University, Seoul 05006, Korea; 4 Department of Electronics and Communication Engineering, University Institute of Technology, Burdwan 713104, India; 5 Department of Artificial Intelligence, Sejong University, Seoul 05006, Korea

**Keywords:** metamaterial, microstrip antenna, gain, dual band frequencies, AMC backing structure, wireless communication

## Abstract

This work presents the design and fabrication of a metamaterial-based stimulated dual band antenna on FR4 material (dielectric constant 4.3) to operate in Industrial, Scientific and Medical (ISM) and Radio-frequency Identification (RFID) applications. The antenna model had an overall dimension of 70 × 31 × 1.6 mm^3^ with etched T-slots and L-slots for dual band resonance. The main objective of this work was to enhance the gain performance characteristic at the selected dual band frequencies of 0.915 GHz and 2.45 GHz. Initially, it achieved a narrow bandwidth of 0.018 GHz with a gain of 1.53 dBi at a lower frequency, and 0.13 GHz of bandwidth featuring 4.49 dBi of gain at a higher frequency. The antenna provided an impedance bandwidth of 2% (0.905–0.923 GHz) and 5% (2.382–2.516 GHz) at two resonating frequencies. The antenna was integrated with a designed novel AMC structure to enhance the gain in CST Microwave Studio software with the finite integration method. The characteristic features of the AMC unit cell were observed at 0.915 GHz and 2.45 GHz frequencies and after antenna integration, the final prototype achieved a gain of 2.87 dBi at 0.915 GHz and 6.8 dBi at 2.45 GHz frequencies.

## 1. Introduction

Modern technology advancements have increased the technical requirements for RFID and ISM band systems in several sectors. The RFID technologies have proven quite popular in many industries, including manufacturing, retail, supply chain, and transportation, whereas ISM band technology is the principal band used by residential users and commercial businesses for WiFi, Bluetooth, cordless phone, printer, keyboard, mouse, and game controller applications. The ultra-high frequency (UHF) RFID tag operates in the 869 MHz-928 MHz frequency range. The Federal Communications Commission (FCC) assigns different frequencies for various purposes. The FCC reserves the 2400 and 5000 MHz frequency bands in the United States for unlicensed Industrial, Scientific and Medical (ISM) applications. The use of ISM equipment causes electromagnetic interference, which disrupts radio communications on the same frequency. As a result, this technology was limited to specified frequency ranges. 

Metamaterials are artificial materials with a more compact size than conventional material structures and have some infrequent properties, like a negative refractive index, negative permeability, double negative characteristic, etc., which do not exist in natural materials [1]. The basic unit elements of metamaterials for improving antenna performance are the Split Ring Resonator (SRR), Complementary Split Ring Resonator (CSRR), and Electric inductive Capacitive (ELC). The SRR and CSRR structures inspired by metamaterials have been used as radiating elements to improve antenna radiation characteristics. Metamaterials are in high demand for the purpose of providing good gain and radiation efficiency due to properties, such as negative permittivity, negative permeability, and negative refractive index of the material. In recent years, some standard electromagnetic simulation techniques have been proposed for antenna engineering enhancements, such as highly reflective index, metal-dielectric metal (MDM), surface plasmon resonance absorber, double plasmon induced transparencies in aperture-coupled metal-dielectric-metal (MDM), and highly sensitive refractive index based on metamaterial [2]. Many researchers have focused on split-ring structures to improve bandwidth. Antennae using metamaterial with miniaturization techniques having a square split ring resonator (SRR) and wire strip have been proposed that operate between 1 GHz and 4 GHz frequency for Wi-max and mobile communication applications [3]. A simple structure with a metamaterial-inspired antenna covering the dual band at 2.5 GHz and 3.5 GHz can be used for wireless applications [4]. The concept of Complementary Split Ring Resonator (CSRR) was explored to get a dual band ranging with first band (1.61–1.84 GHz) resonating at 1.72 GHz and second (2.08–2.5 GHz) at 2.17 GHz with substrate dimensions of 50 × 50 × 1.6 mm^3^ for mobile applications and wireless standards for input reflection coefficient and bandwidth improvement due to the presence of partial ground plane [5]. The C-shaped patch loaded with a dipole antenna was proposed, which provided dual band characteristics at UHF band (850 MHz–930 MHz) and ISM band (2.41 GHz–2.54 GHz) for RFID applications [6]. The Stealth capability of the dual band antenna was improved by loading a metamaterial absorber in the antenna by reducing the Radar Cross Section (RCS) [7]. A CPW-fed line with trapeziform ground plane and tapered impedance transformer line was employed with compactness 31.7 × 27 × 1.6 mm^3^ covering between (2.595–2.654 GHz) and (3.185–4.245 GHz) to improve the antenna’s impedance matching, that can be applicable for wireless communication systems [8]. A dual band AMC-backed miniaturized antenna was designed for ISM frequency bands with dimensions 28.81 × 19.22 × 1.58 mm^3^ at 2.45 and 5.8 GHz [9]. A dual band metamaterial-inspired antenna shows reliable impedance bandwidth, radiation pattern and gain compatible for Monolithic microwave circuit (MMIC) technology [10]. In [11], a bowtie dual band antenna loaded by AMC ground plane was used for RFID applications. In combination with SRR, co-polarization and cross polarization of radiation patterns produce dual band characteristics [12]. Conventional microstrip patch antennas are limited by low gain and low efficiency, but short pins can overcome these limitations [13]. In [14], the proposed antenna was designed to reside in the frequency bands (860–960 and 902–928 MHz) for UHF RFID applications. A split ring resonator, which is metamaterial having two concentric metallic rings with splits in both rings, was a key element to achieve antenna miniaturization [15]. A rectangular microstrip antenna with an inverted E-slot and U-slot having the size 35.05 × 18.24 × 1.6 mm^3^ offering improved gain, radiation efficiency with low cost, controlled radiation characteristics, and with a single layer design suitable for S-band, C-band, and wireless applications was reported in [16]. By using an Electric-inductive- Capacitive (ELC) resonator, a dual band with improved return loss characteristics can be obtained along the E-plane and H-plane and negative permeability property of metamaterial was described in [17]. The physical parameters of the ground plane width and split gap of the SRR were varied to achieve optimum resonance with size 31 × 25 × 1.6 mm^3^ for WiMAX and WAVE applications [18]. A dual band microstrip antenna with two electrically coupled patches operating at 38 GHz and 60 GHz exhibited good performance regarding the output parameters, like gain bandwidth, radiation pattern, and efficiency, and is suitable for 5G mobile communication with excellent diversity schemes [19]. A MIMO-based Dielectric Resonator antenna (DRA) resonating at 28 GHz and 38 GHz reduces coupling without affecting the key parameters like gain, impedance matching, radiation pattern, efficiency, and is suitable for 5G mobile handset [20]. An impedance matched electronic circuit-based absorber was designed for the enhancement of gain in 5G applications. This metamaterial absorber resonated at 24 GHz and 28 GHz frequencies, based on the total inductance given by the meander line structure [21]. A low-profile wideband AMC-backed log-periodic meandered dipole array (LPMDA) antenna with improved gain and constant wideband characteristics was reported for communication system applications in UAVs and aircraft [22]. A dual band monopole antenna and a polarization rotation artificial magnetic conductor (PRAMC) was enabled, backward radiation was alleviated, and dyadic reflection coefficient analysis was employed to understand the operating mechanism, which is suitable for Wi-max and ISM applications [23]. The performance comparison of different compact antennas (conventional, EBG-based, and SRR-based) along with specific absorption rate (SAR) analysis suggests that they are well-suited for biomedical sensors, to work at 2.4 GHz and 5.8 GHz dual band frequencies [24]. A fully textile dual band logo antenna integrated with a reflector for application in IoT wearable devices achieves improvement in radiation, robustness, and efficiency both in free space and on humans, notebook, paper, and laptop phantom at dual band frequencies [25].

In this paper, a proposed antenna was designed with dimensions of 70 × 31 × 1.6 mm^3^ by FR4 substrate which has good resistance. As compared with other models, which resonate at higher frequencies, the proposed model resonates at 0.915 GHz and 2.45 GHz. The resonating frequencies are in a lower band of frequencies, which are used for L and S band applications. This makes the proposed antenna highly effective for RFID and ISM band-based systems. Additionally, the proposed antenna provides 0.018 GHz and 0.13 GHz bandwidths for dual band frequencies. The attractive feature in the designed antenna is enhancement of gain as compared with the others reported in literature.

### Purpose of the Design

In contrast to the typical antennas, metamaterial antennas can reduce the number of elements, size miniaturizing, and enhance their characteristics. As a result, they are widely used in the wireless communication industry to enhance the performance of various applications. Computer software technology (CST) simulated the proposed antenna array configuration. Metamaterials are mainly made up of artificial materials with different characteristics not found in natural materials. As a result, their unique properties have led to their becoming a research focus. Metamaterials are generally called “Left-handed materials” or the “negative-index materials” [26]. Metamaterials are divided into different classes: negative refractive index, single negative, hyperbolic, band gap, double positive medium, chiral, and Frequency selective surfaces (FSS). The FSS can exhibit subwavelength characteristics, sometimes called Artificial Magnetic Conductor or High Impedance surfaces. Frequency selective surfaces (FSSs) implemented on single-layer or stacked printed circuit boards (PCBs) have found multiple applications in the last decade as randoms, absorbers, polarizers, artificial magnetic conductors, spatial filters and shields, dichroic reflectors and reflectors for antenna gain enhancement, etc. [27]. Many researchers have used FSS array unit cells as a reflector by printing them on a dielectric substrate on one side of the sheet to enhance the gain over frequencies with varying enhancements [28]. 

A monopole antenna was developed and fabricated for ISM and RFID band applications in the proposed work. The antenna was modelled to operate at the dual band and simulated using CST software. Unit cell structure was developed that exhibited negative permittivity and negative permeability characteristics at two frequency bands. An antenna was placed over an AMC structure to enhance gain. The AMC structure consisted of the 3 × 3 array periodic unit cell. The antenna with the AMC backing operated at two frequencies. The structural features of the proposed antenna, such as the reflection coefficient, bandwidth, gain, and radiation patterns, were studied for the antenna design.

Section 2 describes the antenna design, materials used, iterations, and the AMC backing structure design. Section 3 describes the parametric results of the antenna without and with the AMC Backing. Section 4 compares the proposed antenna’s performance with the existing literature. Finally, Section 5 describes the conclusion of the work.

## 2. Materials and Methods

### 2.1. Antenna Design

**Figure 1:**
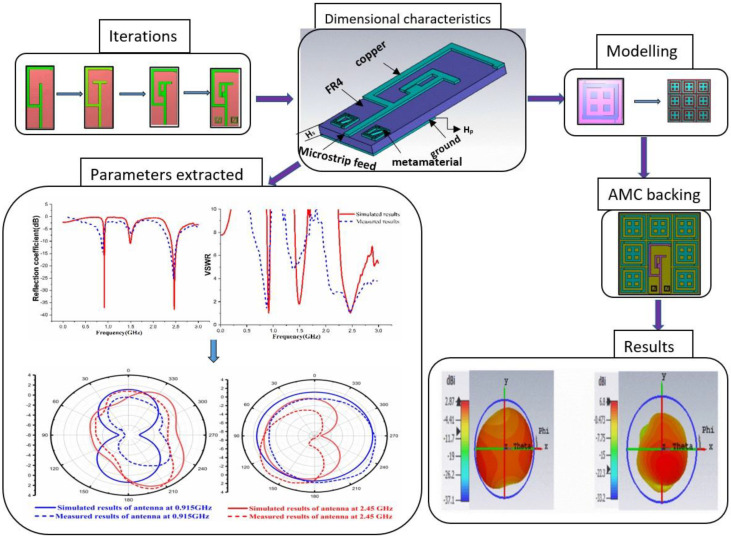
Overview of Design, Modelling analysis.

Figure 1 illustrates the overview of the antenna design and modelling characteristics and associated analysis. A monopole antenna was designed on FR4 material with a dielectric constant of 4.3 and a thickness of 1.6 mm. The patch was designed with copper material with a thickness of 0.035 mm and a partial ground on the opposite side of the antenna. Further, a microstrip feedline was provided for the patch. Impedance matching was considered to feed the patch and the 50 Ω probe line. Metamaterials were placed on both sides of the feed line. The fabrication was performed for the antenna with and without the AMC structure. 

### 2.2. Iterations of the Designed Antenna

**Figure 2:**
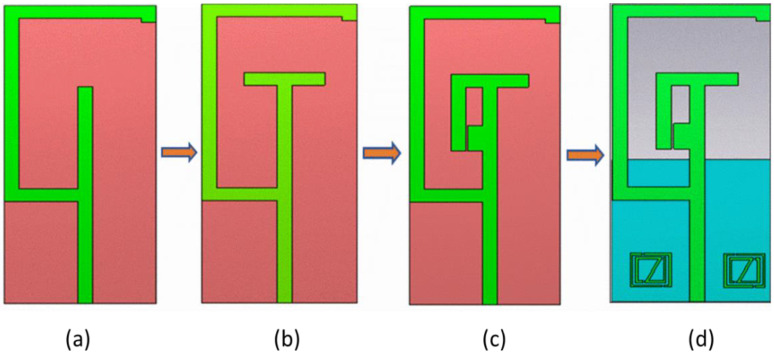
Antenna Iterations, (**a**) iteration 1, (**b**) iteration 2, (**c**) iteration 3, (**d**) iteration 4.

The proposed antenna in the design stage underwent different iterations to operate at dual band characteristics, as illustrated in Figure 2. The fundamental iteration of the microstrip patch antenna started with the design of the L-shaped stub attached to the microstrip feed line, as depicted in Figure 2a. Subsequently, the iteration was modified with the addition of T-shaped stub to obtain narrow BW, as shown in Figure 2b. The antenna was altered by placing another two stubs with the T-shaped stub, as presented in Figure 2c. The final antenna was restructured by positioning the metamaterials on both sides of the feed line with a partial ground on the other side of the substrate as illustrated in Figure 2d. The metamaterial arrangement consisted of double split ring resonators with a gap in between the resonators.

**Figure 3:**
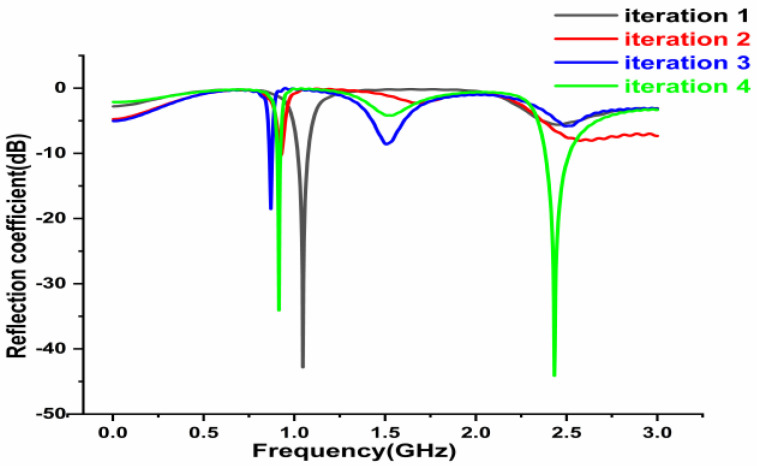
Results of antenna iterations.

Figure 3 highlights the results of iterations of the antenna. The black line depicts the effect of iteration 1, in which the antenna resonates at a single frequency at 1.1 GHz with S_11_ value of −43 dB. The red line shows the result of iteration 2, which specifies that the antenna radiates at a dual band with less reflection parameter while, iteration 3 of the antenna is indicated by the blue line, which operates in a dual band but with less S_11_ values at 0.85 GHz and 1.6 GHz frequencies. Iteration 4 is indicated in the green line, wherein a successful dual band is observed with the characteristics of the S_11_ value of −35 dB at 0.915 GHz and −44 dB at 2.45 GHz frequencies, respectively.

### 2.3. Design Principle of Proposed Antenna

**Figure 4:**
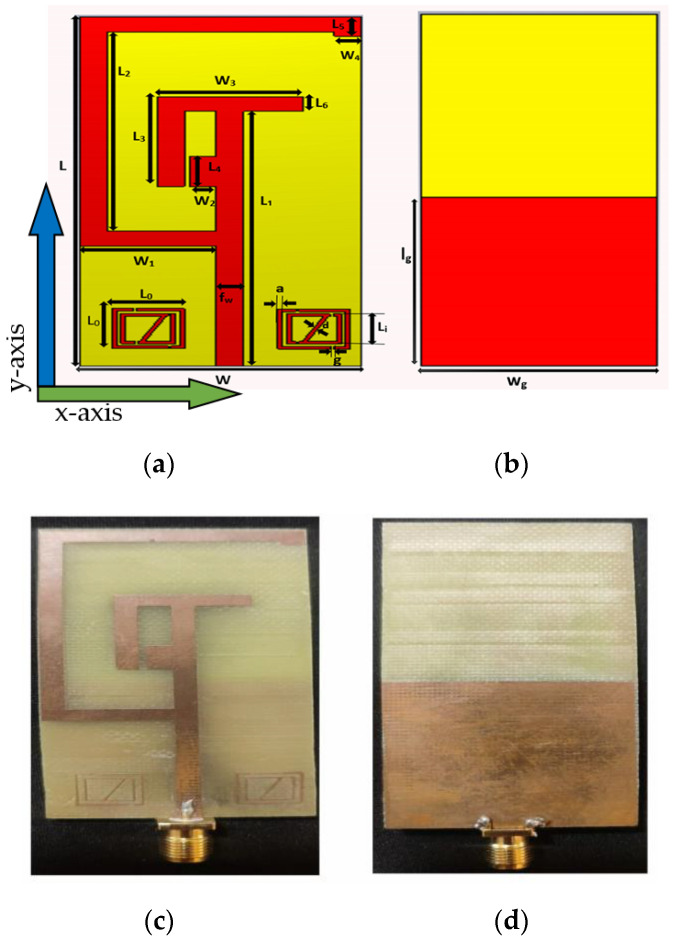
Designed Antenna (**a**,**b**), Fabricated Antenna (**c**,**d**), (**a**,**c**) Front view, (**b**,**d**) Rear view.

Computer Simulation Technology (CST) software is being widely employed to interrogate and simulate the prototype antenna. The antenna design was developed upon a substrate of FR4 material with 4.3 dielectric constant with thickness H_t_, 1.6 mm. Thus, the size of the proposed monopole antenna was formed with dimensions of 70 mm × 31 mm. The width of the microstrip transmission line was f_w_ = 3 mm. The dimensional characteristics of the proposed monopole antenna are depicted in Figure 4. The antenna front view and the rear view are shown in Figure 4a,b. The prototype fabricated antenna is illustrated in Figure 4c,d.

The specifications of the designed antenna parameters are listed in Table 1.

On both sides of the feed line a metamaterial was positioned. Metamaterials are designed with the two squared split ring resonators. A gap of 0.4 mm separated the outer and inner split resonators. A diagonal metal strip connected the inner split resonator from one end to the other. The length of the outer rectangle of the metamaterial L_0_ = 8 mm. L_i_ = 7.2 mm is the inner rectangle of the metamaterial, while l_g_ = 33.5 mm specified the partial ground length, and w_g_ = 31 mm specified the ground width. Split ring resonators on both sides of feed line consisted of two square-shaped concentric metal rings separated by a gap and splits on either side of the two square-shaped rings. The gap between the inner and outer rings along with the splits at the rings induce Magnetic Resonance. For the lower band, the design used an outer square metallic strip forcing the patch to radiate an equivalent magnetic-current loop. For the upper band, another magnetic current loop was created by adding a metamaterial structure near the feed line on the patch. By placing split ring resonators, antenna exhibits narrow dual band frequency characteristics. This design was selected as it was compact in size, low cost and achieved desired bandwidth accurately.

Depending upon the fundamental equations, the mathematical formulae [29] to design the antenna can be derived as follows:

The operating wavelength of the antenna *λ*_0_ is calculated by

*λ*_0_ = *c*/*f_r_* (1)

where *f_r_* is the resonant frequency; *c* is the value of the velocity of light in free space.

Wavelength is calculated by the formula

(2) λd=λ0εr

where *ε_r_* is the dielectric constant.

The thickness of the substrate, *H_t_* is given by

(3) $$H_{t}\leq \frac{0.3\times c}{2\pi f_{r}\sqrt{\varepsilon_{r}+1}}$$

The microstrip feed line width is given by

(4) $$F_{w}=\frac{c}{2f_{r}}\sqrt{\frac{2}{\varepsilon_{r}+1}}$$

The effective dielectric constant, *ε_reffective_* is given by

(5) $$\varepsilon_{reffective}=\left(\frac{\varepsilon_{r}+1}{2}\right)+\left[\left(\frac{\varepsilon_{r}-1}{2}\right){\left(1+12\frac{h}{w}\right)}^{-1/2}\right]$$

The Effective length is given by

(6) $$L_{eff}=\frac{c}{2f_{r}\sqrt{\varepsilon_{e}}}$$

The Effective width of the antenna is given by

(7) $$W_{eff}=\frac{c}{2f_{r}}\sqrt{\frac{2}{\varepsilon_{r}+1}}$$

### 2.4. Unit Cell Design and Parameter Specifications

**Figure 5:**
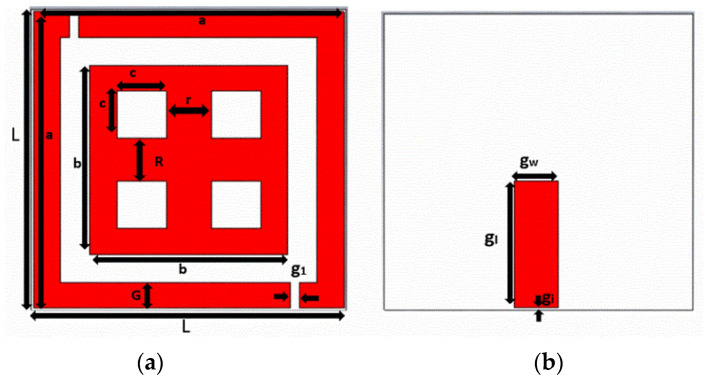
Unit cell Structure, (**a**) Top view, (**b**) Rear view.

A unit cell was designed and simulated by using CST software. The unit cell was designed to have permittivity and permeability in negative characteristic features. The designed unit cell consisted of double squared-shaped split ring resonators in which the outer ring splits on either side while the inner one was closed. Two perpendicular metal strips connected the opposite side of the closed inner ring resonator. A unit cell is generally lesser than 1/10th of the resonating wavelength. The proposed dual band unit cell dimensional view is depicted in Figure 5, and the parametric specifications are listed in Table 2.

**Figure 6:**
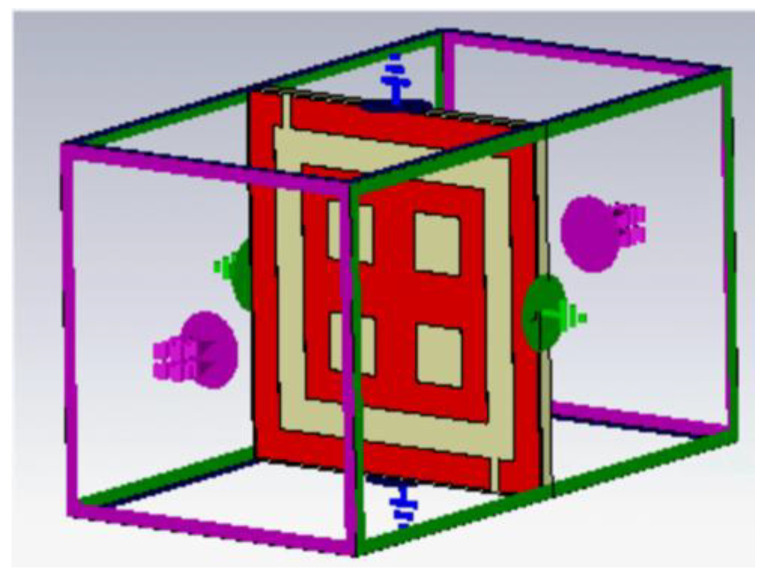
Boundary conditions of the designed unit cell.

Figure 6 portrays the boundary conditions of the unit cell. Such an arrangement facilitates a waveguide port, perfect electric conductor (PEC), and perfect magnetic conductor (PMC) to set perfectly on the *x*-axis, *y*-axis, and *z*-axis, respectively. The incident field propagates along the x direction with E and H fields along the y and z directions.

**Figure 7:**
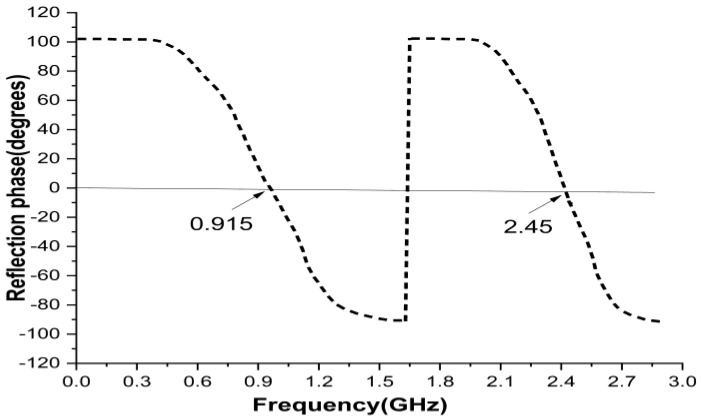
Reflection phase results of unit cell.

The reflection phase results are shown in Figure 7. It can be noticed that the reflection becomes zero twice in the frequency band of interest. At low frequency, the resonance was due to the outer ring of the unit cell. At high frequency, the resonance was due to the plus-shaped structure present inside the inner ring of the unit cell. The two different null reflected phase frequencies can be independently adjusted which is highly suitable for dual band design.

**Figure 8:**
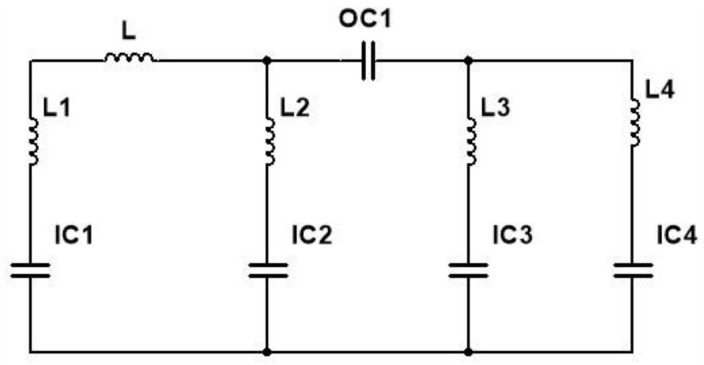
Equivalent circuit of the unit cell.

A wide range of metamaterial patterns have been introduced, viz., double split ring resonator, single split ring resonator (SRR) and the electric ring resonator [30]. Out of all such designs, the split ring resonator is the most used pattern due to its simple geometry. When SRR is excited with a gap perpendicular to an electric field, the resonator exhibits capacitive-inductive resonance that arises from the accumulation of charge at SRR gap area and the current at the side ring of the SRR. The suggested metamaterial had both capacitive and inductive elements. All metal bars and strips of rings acts as inductors, while the spaces between the metal bars or rings serves as capacitors. As a result of this, metamaterial unit cell created an LC resonance circuit. Figure 8 represents the equivalent circuit of the proposed unit cell structure; OC is the outer square ring and IC is the inner squared ring; L is the inductance produced in the outer squared ring and the parameters L1, L2, L3, L4 are the inductance produced in the inner squared ring of the unit cell.

The parametric values of the inductor of designed unit cell structure are L = 6.2 nH, L1 = 220 µH, L2 = 1.1 nH, L3 = 2.2 nH, L4 = 4.3 nH, and the capacitance are sequentially, OC1 = 5.6 pF, IC1 = 0.51 pF, IC2 = 0.15 pF, IC3 = 0.20 pF, IC4 = 1.5 pF. 

The neural impedance of the FR4 substrate can be calculated [31] as

(8) $$Z_{sub}=\frac{z_{0}}{\sqrt{\varepsilon_{r}}\;}\;=\frac{50}{\sqrt{4.3}}$$

According to the strip line theory [32], the inductance of the strips line is calculated using

(9) $$L\approx 2\times 10^{-4}l\left[ln\left(\frac{l}{w+t}\right)+1.193+0.2235\left(\frac{w+t}{l}\right)\right]$$

where ‘*l*’ is the length of the strip line, ‘*w*’ is the width and ‘*t*’ is the thickness. 

The capacitance per unit length of the paralleled strip lines is calculated using

(10) $$C\approx \varepsilon_{e}\varepsilon_{0}F\left(k\right)$$

where ‘$\varepsilon_{e}$’is the permittivity of the free space, ‘$\varepsilon_{0}$’ and *F*(*k*) are calculated as

(11) $$\varepsilon_{e}=1+\left(\varepsilon_{r}-1\right)F\left(k\right)∕2F\left(k1\right)$$

(12) $$F\left(k\right)=\{\begin{array}{c} \frac{1}{\pi }ln\left(2\frac{1+\sqrt{k^{\prime }}}{1-\sqrt{k^{\prime }}}\right),0<k\leq \frac{1}{\sqrt{2}}\\ \pi ln\left(2\frac{1+\sqrt{k^{\prime }}}{1-\sqrt{k^{\prime }}}\right),\;\frac{1}{\;\sqrt{2}}<k\leq 0 \end{array}$$

where $k=\frac{a}{b}$, $k^{\prime }=\sqrt{1-k^{2}}$, $k1=\sin h\left(\frac{\pi a}{2h}\right)/\sin h\left(\frac{\pi b}{2h}\right)$.

To analyse the structural characteristics of the proposed unit cell, the reflection (*S*_11_) and transmission (*S*_21_) coefficients are obtained to calculate the permittivity and permeability [33] and are expressed as follows

*V*_1_ = *S*_11_ + *S*_21_ (13)

*V*_2_ = −(*S*_11_ − *S*_21_) (14)

$$\varepsilon_{r}\approx \frac{2}{jk_{0}d}\times \frac{\left(1-v_{1}\right)}{\left(1+v_{1}\right)}$$

Effective permittivity

(15) $$\varepsilon_{r}\approx \frac{c}{j\pi fd}\times \left\{\left(1-s_{11}-s_{21}\right)/\left(1+s_{11}+s_{21}\right)\right\}$$

$$\mu_{r}\approx \frac{2}{jk_{0}d}\times \frac{\left(1-v_{2}\right)}{(1+v_{2)}}$$

Effective permeability

(16) $$\mu_{r}\approx \frac{c}{j\pi fd}\times \{\left(1-s_{21}+s_{11}\right)/\left(1+s_{21}-s_{11}\right)\}$$

where *k*_0_ = *ω*/*c*, *d* = slab thickness, *c* = speed of light.

**Figure 9:**
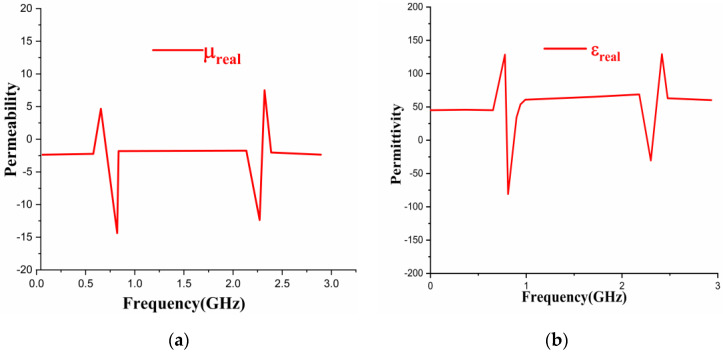
Results of Unit Cell Structure, (**a**) Permeability, (**b**) Permittivity.

The unit cell structure was designed on an FR4 substrate, and results were observed using CST software. The electromagnetic characteristics can be well explained by reflection (*S*_11_) and transmission (*S*_21_) coefficients. The simulated results of effective permittivity (*ε_real_*) and permeability (*µ_real_*) are observed in Figure 9. However, Figure 9a shows the permeability of the metamaterial having negative values at 0.915 and 2.4 GHz frequencies and Figure 9b shows negative permittivity values at 0.915 and 2.4 GHz frequency bands.

### 2.5. AMC Structure Design

The AMC structure is a class of metamaterial that has a 0° phase reflection to an incident phase upon the surface. It is a variant of the Perfect Electric Conductor (PEC), which has a −180° phase to an incident wave. The AMC region is usually accepted when the reflection phase is 0 ± 90° for the range of frequencies. 

**Figure 10:**
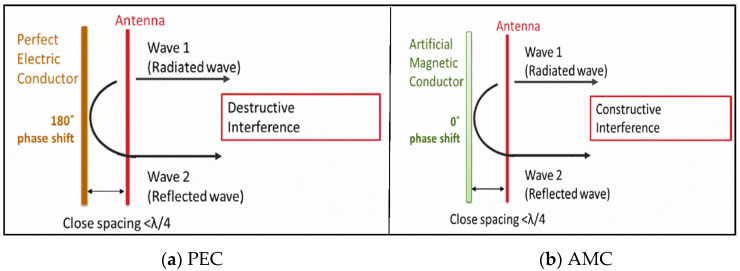
An Antenna placed flat against the PEC and AMC.

Figure 10 exemplifies the antenna close to the PEC and AMC structure. When an antenna was placed in front of the PEC with space less than λ/4, its 180° phase shift caused destructive interference, which results in poor reflection coefficient value and low total efficiency. Alternatively, when the antenna was placed in front of the AMC structure at spacing less than λ/4, its 0° phase shift caused constructive interference, resulting in good return loss and sufficient bandwidth [34].

**Figure 11:**
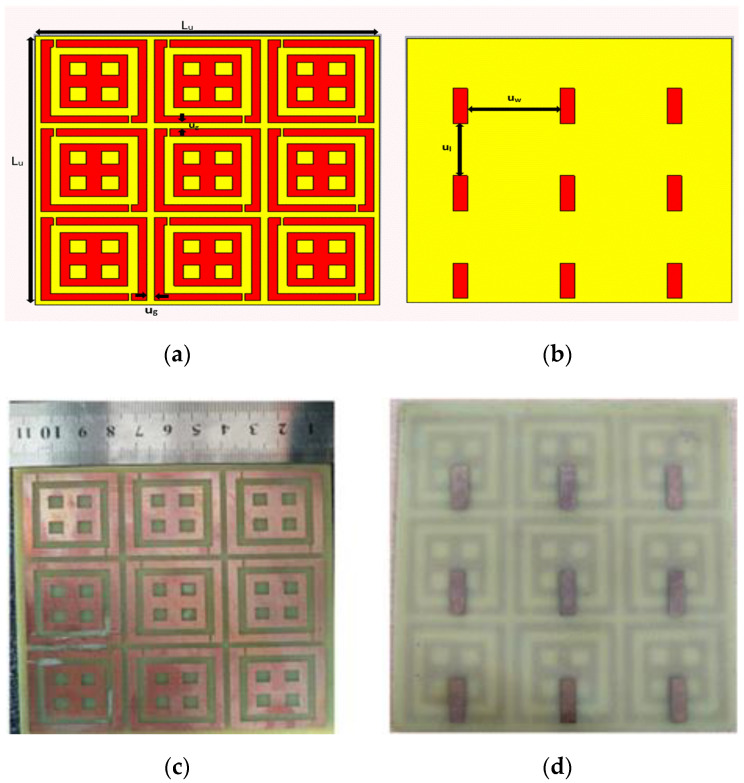
3 × 3 Array Structure, Proposed 3 × 3 Array (**a**,**b**), Snapshot of Fabricated 3 × 3 Array. (**c**,**d**), (**a**,**c**) Front view, (**b**,**d**) Back view.

The unit cell was arranged periodically in a 3 × 3 array. Figure 11 portrays a 3 × 3 periodic array structure of the designed unit cell. The design geometry is shown in Figure 11, and the parameter specifications are listed in Table 3 wherein L_u_ represents the length of the unit cell’s periodic array. The array’s thickness was considered to be 1.6 mm and gap between the unit cell was 2.5 mm. The design consists of rectangular slots in the ground, and u_w_, u_l_ are the space gaps between the slots. Figure 11a,b presents the front and rear views of the periodic array. Figure 11c,d depicts the array’s front and back views of the fabricated unit cell structure.

**Figure 12:**
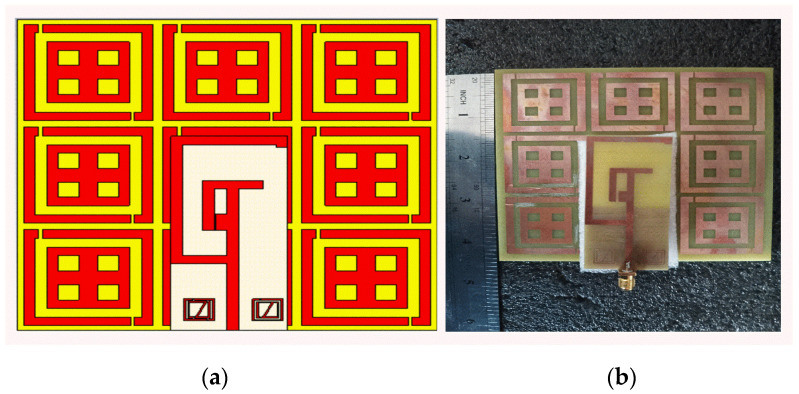
Prototype AMC Backing Antenna (**a**) Antenna top view (**b**) Fabricated Antenna.

The designed antenna was placed against the AMC with a spacing of a 25 mm gap. Figure 12a illustrates the front view of the AMC structure. Figure 12b is the true image of the fabricated AMC Backing antenna.

## 3. Results

### 3.1. Results of the Antenna without AMC Backing

**Figure 13:**
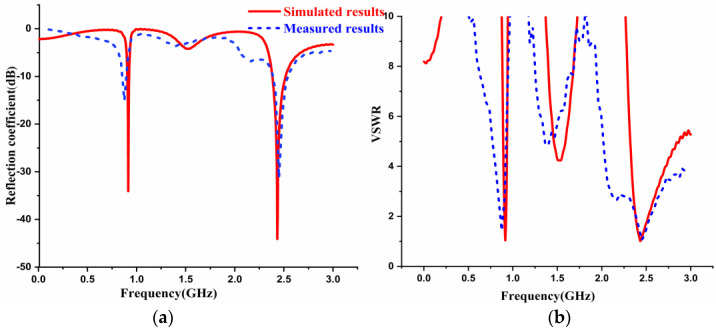

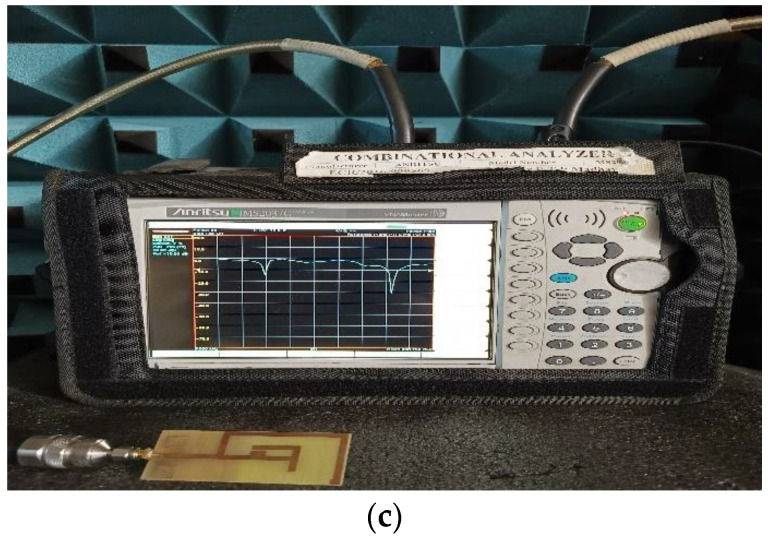
Simulated and Measured Results of Antenna without AMC Backing (**a**) Reflection Coefficient, (**b**) VSWR, (**c**) Snapshot of antenna results using VNA.

A prototype antenna was being designed and developed. The true image and structure is represented in Figure 3c,d. The antenna without the AMC backing structure was designed in FR4 material and simulated using CST software. The simulated and fabricated results of S-parameters and VSWR of the antenna without AMC construction is shown in Figure 13a,b. The antenna without the AMC Backing exhibited resonance peaks at 0.915 GHz and 2.45 GHz that cover the bandwidth of 20 MHz and 130 MHz for simulation. The measured values exhibit 20 MHz and 130 MHz bandwidth at 0.912 GHz and 2.45 GHz frequency bands. Hence, the antenna is applicable for RFID applications (902~928 MHz) as well as WiMAX (2.31~3.49 GHz) and Bluetooth (2.346~2.906 GHz) applications. Figure 13c shows a photograph of measured values of the prototype antenna using VNA.

**Figure 14:**
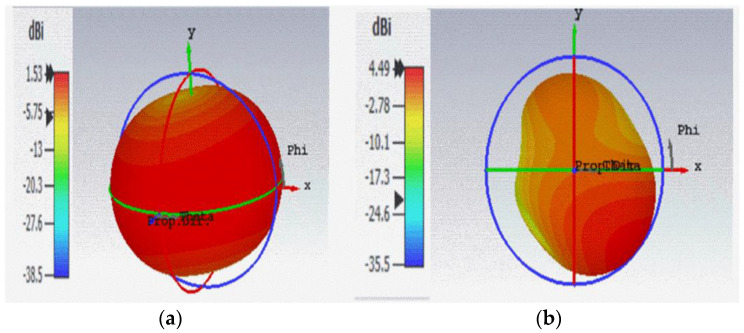
Simulated Gain of Antenna without AMC Backing (**a**) at 0.915 GHz (**b**) at 2.45 GHz.

The simulated gain of the antenna in the absence of AMC Backing is represented in Figure 14. In such a situation, it was detected at 0.915 GHz and 2.45 GHz frequencies as 1.53 dBi and 4.49 dBi, respectively. The simulated bandwidth obtained at the lower frequency band (0.905~0.923 GHz) was 0.018 GHz, and at the higher frequency band (2.382~2.516 GHz) it was 0.13 GHz. Further, it was observed that, the square metallic patch close to the feed line increased the performance at high frequency.

**Figure 15:**
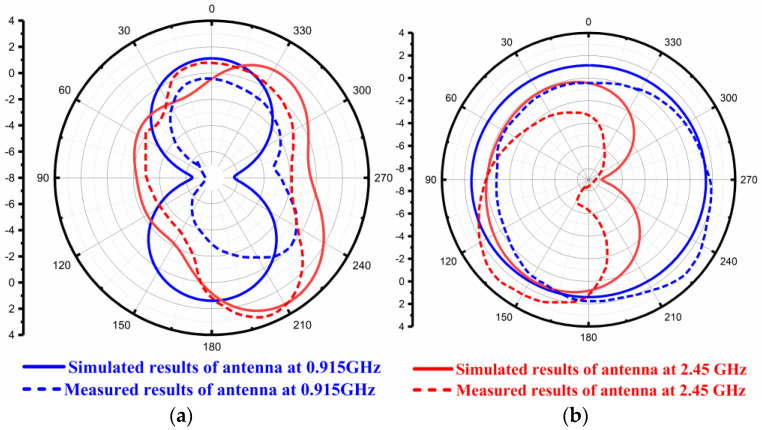
Simulated and Measured Radiation Pattern of the Antenna without AMC Backing. (**a**) Along E-Plane (**b**) Along H-Plane.

At 0.915 GHz and 2.45 GHz frequencies, radiation patterns are depicted in Figure 15. Figure 15a represents the radiation profiles which were obtained by simulation and fabrication process along the E-plane at two resonating frequencies, i.e., at 0.915 GHz and 2.45 GHz. Figure 15b represents the simulated and measured results of radiation patterns in the H-plane at 0.915 GHz and 2.45 GHz frequencies. The blue line represents the radiation pattern at 0.915 GHz, while the red line shows the radiation pattern at 2.45 GHz frequency bands. It is evident that the simulated radiation patterns at 0.915 GHz along the E-Plane had a bi-direction nature, and along the H-Plane, it was omnidirectional. The simulated radiation patterns at 2.45 GHz along the E-Plane was a dipole, and along H-Plane it was nearly omnidirectional.

### 3.2. Results of the Antenna with AMC Backing

**Figure 16:**
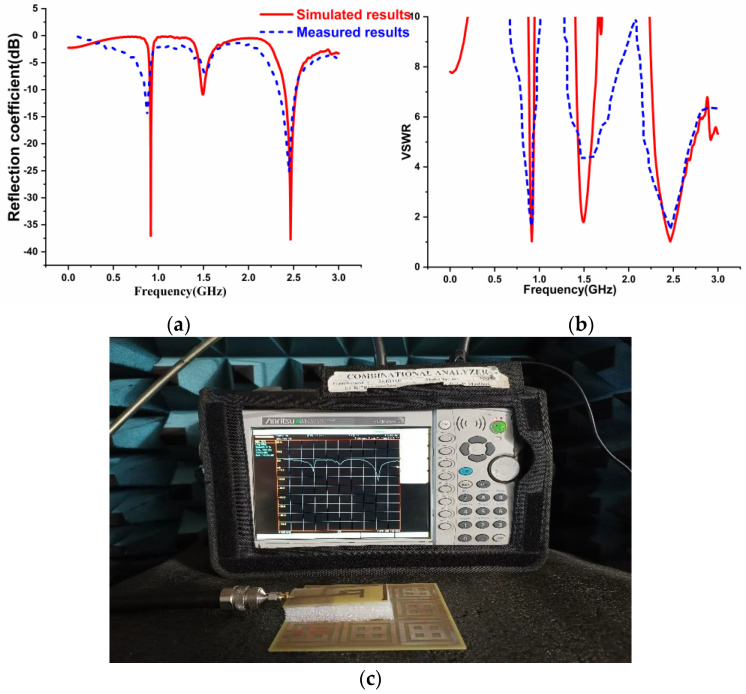
Simulated and Fabricated results of the antenna with AMC Structure (**a**) Reflection Coefficient (**b**) VSWR (**c**) Snapshot of the antenna with AMC Backing Results.

The simulated and fabricated reflection coefficient and VSWR outcomes of the prototype design are shown in Figure 16. The antenna was placed by spacing a 25 mm gap against the periodic array of the unit cell. Figure 16a illustrates the S-parameter of the antenna with the AMC backing. It was observed that the antenna with the AMC backing provides dual band frequencies at 0.915 GHz and 2.45 GHz. Figure 16b represents the VSWR result of the antenna with AMC backing. Figure 16c depicts the photograph of the antenna results using VNA with the AMC Backing.

**Figure 17:**
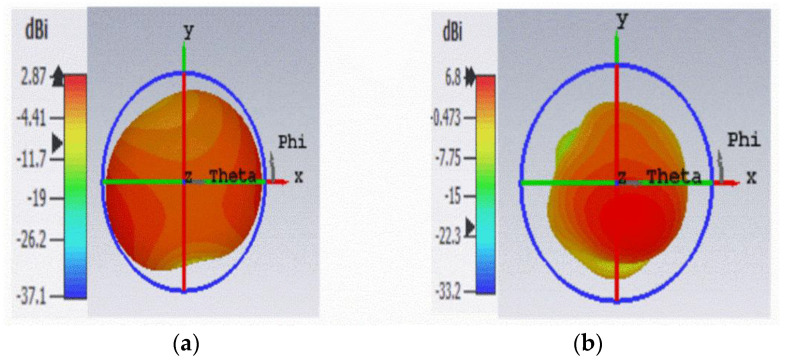
Simulated Gain of Antenna with AMC Backing, (**a**) at 0.915 GHz, (**b**) at 2.45 GHz.

The simulated bandwidth obtained at 0.915 GHz and 2.45 GHz frequencies, when the antenna was placed in front of the AMC structure, was (0.902~0.920 GHz) 0.018 GHz, and (2.378~2.508 GHz) 0.13 GHz. Figure 17a,b illustrates the simulated gain results at 0.915 GHz and 2.45 GHz frequencies for the antenna with the AMC backing. The simulated gain obtained at 0.915 GHz was 2.87 dBi and at 2.45 GHz was 6.8 dBi. The most attractive part of this AMC structure was that it enhanced the gain without influencing the antenna’s bandwidth.

The simulated results of the antenna show that the gain had improved from 1.53 dBi to 2.87 dBi at 0.915 GHz, and 4.49 dBi to 6.8 dBi at 2.45 GHz, without affecting the bandwidth of the antenna, both with and without the AMC backing.

**Figure 18:**
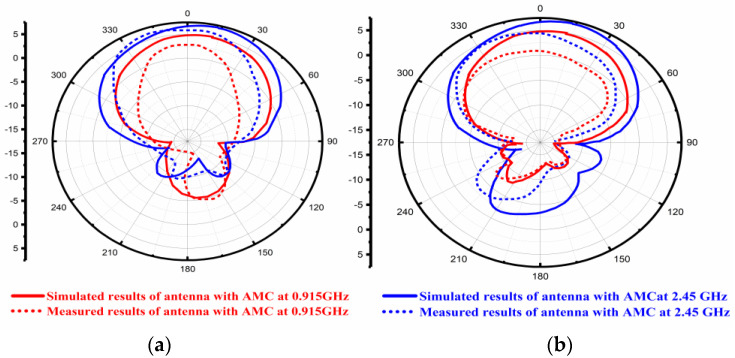
Simulated and fabricated Radiation Pattern of AMC backing Antenna (**a**) Along the E-Plane, (**b**) Along the H-Plane.

Figure 18a,b shows the radiation pattern of the prototype antenna with the AMC backing. The radiation patterns along the E and H planes were observed at 0.915 GHz and 2.45 GHz frequencies. The red line depicts the radiation pattern of 0.915 GHz along the E-Plane and the H-Plane while, the blue line depicts the radiation pattern at 2.45 GHz along the E and H-Plane. The proposed antenna with the AMC backing exhibited the desired directional radiation pattern characteristics along both planes at dual operating frequencies. In addition to the above, it was noticed that there was a good agreement between the simulated and measured outcomes.

**Figure 19:**
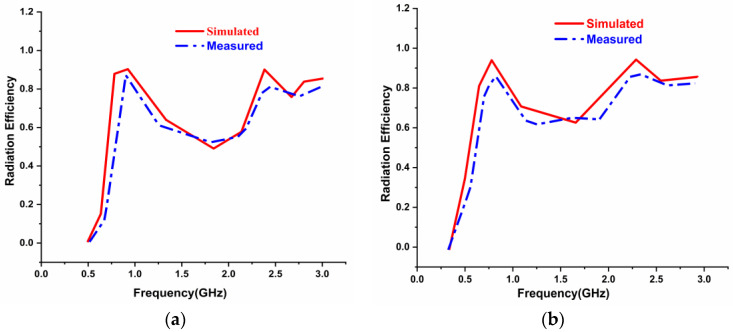
Radiation Efficiency results of the antenna (**a**) without the AMC Backing (**b**) with the AMC Backing.

The radiation efficiency plots concerning the frequency in GHz of the antenna with and without the AMC Backing is presented in Figure 19. The red and blue lines depict simulated and measured results, respectively. It was observed that 93% and 90% radiation efficiencies were obtained at 0.915 GHz and 2.45 GHz frequency without the AMC Backing. When the AMC was loaded, radiation efficiencies were increased to 96% and 93%, respectively, at lower and higher frequency bands.

**Figure 20:**
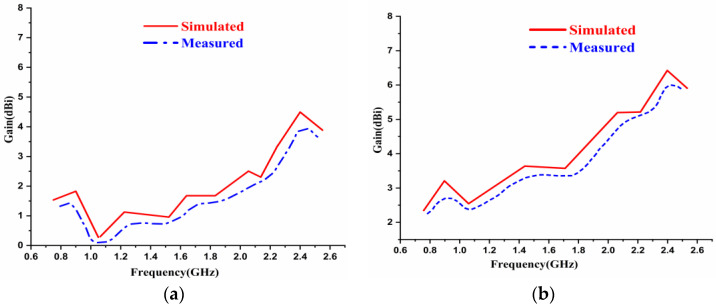
Gain versus Frequency plot of the antenna (**a**) without AMC Backing (**b**) with AMC Backing.

The simulated and measured gain versus frequency results of the antenna with and without the AMC Backing is depicted in Figure 20. Figure 20a portrays the gain versus frequency plot without the AMC while, the red lines illustrate the antenna’s simulated results, and the blue lines depict the measured results. Figure 20a shows that the gain at the operating frequency was 1.83 dBi at 0.915 GHz and 4.38 GHz at 2.45 GHz frequencies, respectively, for the antenna without an AMC-backed structure. From Figure 20b, it can be observed that the gain has an improvement of 4.3 dBi at 0.915 GHz and 6.64 dBi at 2.45 GHz for the antenna with the AMC backed structure. Considering the above, at the operating frequencies of 0.915 GHz and 2.45 GHz, the gain characteristics were increased by placing the antenna in front of the AMC structure. The same is illustrated in Figure 20a,b.

**Figure 21:**
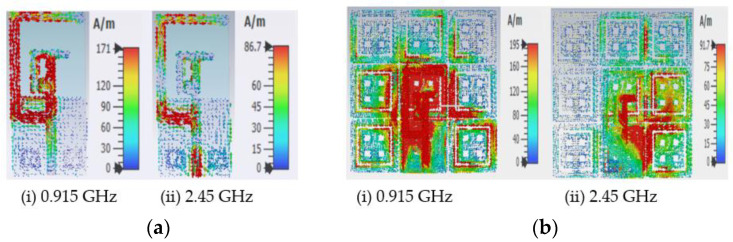
Current distribution characteristics, (**a**) antenna without AMC Backing, (**b**) antenna with AMC Backing.

The surface current distributions of the antenna with and without the AMC Backing are displayed in Figure 21. One can see from Figure 21a that the radiation was achieved more at the L-shaped slot at 0.915 GHz frequency. At 2.45 GHz, the radiation was achieved at the feed for the antenna without the AMC Backing. Figure 21b shows that the current radiated more throughout the antenna at 0.915 GHz. The radiation was believed to be along the T-shaped slot at 2.45 GHz frequency. The antenna radiated more when the antenna was placed above the AMC Backing at two frequency bands rather than when the antenna was without the AMC Backing. 

## 4. Performance Comparison with the Existing Models

The designed antenna was tested for applicability in the desired applications with Vector Network Analyzer in the Anechoic Chamber. The obtained parameters from the proposed antenna were compared with the existing literature. The remarkable values in gain and efficiency were found to be good agreement. The optimized dimensions with the placement of AMC and dual band characteristics with suitable bandwidth are the key features of the designed antenna.

Table 4 shows the performance comparison in which dimension, resonating frequencies, bandwidth, gain, and efficiency of different antennas are compared with the proposed antenna.

## 5. Conclusions

A metamaterial inspired dual band antenna comprising of an AMC Backing, aimed for ISM and RFID applications was sketched, simulated, and fabricated. With the dimensions of 70 × 31 × 1.6 mm^3^, the antenna absorbed relatively little power. The metamaterials used on either side of the antenna created a pathway for it to resonate at two frequencies making it a dual band antenna. According to the simulation results, the metamaterial inspired antenna, performed well at both lower (0.905 ≈ 0.923 GHz) and higher (2.382 ≈ 2.516 GHz) frequencies. The designed antenna provided high gain when placed beneath the AMC Backing structure resulting in a change from 1.53 dBi to 2.87 dBi, and 4.49 dBi to 6.8 dBi, at 0.915 GHz and 2.45 GHz frequencies, respectively. The AMC Backing structure caused the radiation properties of the simulated system to shift from omnidirectional to directional. The AMC loaded antenna structure offered improvement in radiation efficiencies compared to the antenna without the AMC backing. The surface current distributions were observed and discussed for the antenna structures with and without AMC Backing. The parametric properties were obtained with negative values of permittivity, permeability and zero degrees of the reflected phase, making the unit cell perfect. In view of the above, the prototype antenna is an attractive candidate for microwave and UHF bands at Bluetooth, 802.11 wireless network protocol, WIFI, 4G, and LTE applications.

## Figures and Tables

**Table 1:** Parameter Specifications of Proposed Antenna.

Parameters	Dimension (mm)
L	70
W	31
H_t_	1.6
f_w_	3
W_1_	15
W_2_	3
W_3_	16
W_4_	3
L_1_	51
L_2_	40
L_3_	15
L_4_	6
L_5_	3.9
L_6_	3
l_g_	33.5
w_g_	3
L_0_	8
L_i_	7.2
g	0.3
d	0.5
a	0.5

**Table 2:** Parameter Specifications of designed Unit Cell.

Parameters	Dimension (mm)
L	35
a	34.5
b	22
c	5.5
g	1
G	6
r	5
R	5
g_w_	5
g_l_	15
g_1_	0.5
*H_t_*	1.6

**Table 3:** Parameter specifications of 3 × 3 Array periodic structure.

Parameters	Dimension (mm)
L_U_	112
U_g_	2.5
U_w_	32
U_l_	22

**Table 4:** Comparison Table.

Reference No.	Dimension (mm × mm × mm)	Resonant Frequency (GHz)	Bandwidth (GHz)	Gain (dBi)	Efficiency (%)
5	50 × 50 × 1.6	1.7, 2.17	1.61–1.84 2.08–2.5	1.8, 1.6	97.2, 99.1
8	31.7 × 27 × 1.6	2.6, 3.6	2.595–2.654 3.185–4.245	1.4, 1.9	79.3, 95.6
11	140 × 80 × 1.6	1.8, 3.5	0.869–0.8697 0.910–0.914	2.1, 6.74	-
12	27 × 22 × 1.6	2.55, 3.48	2.46–2.64 3.42–3.55	1.42, 0.73	90.66, 70.40
13	30 × 50 × 1.54	1.9, 3.6	1.85–1.93 3.48–3.57	1, 2.4	28, 25
15	21.57 × 25.62 × 1.6	2.36, 4.45	2.29–2.4 3.48–3.57	-	-
16	35.05 × 18.24 × 1.6	3, 4.6	2.28–3.04 4.55–4.65	2.49, 3.68	48.45, 56.52
17	30 × 30 × 0.8	3.74, 5.1	3.57–4.04 4.73–5.59	1.23, 1.57	82, 82
18	31 × 25 × 1.6	3.42, 6.07	3–3.84 5.94–6.25	-	-
24	98 × 109.4 × 0.01	2.4, 5.4	2.39–2.44 5.38–5.43	-	64.7, 52.4
25	45 × 85 × 0.057	2.4, 5.47	2.393–2.488 4.75–6	1.7, 4.5	30, 90
**Proposed work**	70 × 31 × 1.6	0.912, 2.45	0.905–0.923 2.382–2.516	2.87, 6.8	96, 93

## Data Availability

The data presented in this research are available on request from the corresponding author.
